# Environmental and occupational respiratory diseases – 1037. Sensitization to indoor aeroallergens in pediatric patients

**DOI:** 10.1186/1939-4551-6-S1-P36

**Published:** 2013-04-23

**Authors:** Vanessa Yañez-Perez, Sandra Gonzalez-Diaz, Claudia Gallego-Corella, Alfredo Arias, Karla Mejía, Luis Alfredo Dominguez, Maricruz Calva, Lorena Rangel, Hilda Hernández

**Affiliations:** 1 Allergy and Clinical Immunology, Universidad Autonoma De Nuevo Leon - Hospital Universitario “Dr. Jose Eleuterio Gonzalez”, Monterrey, Mexico

## Background

Indoor aeroallergens are the main cause of sensitization. The aim of this study is to identify the most common indoor aeroallergens which tested positive by skin tests in Monterrey, México.

## Methods

A retrospective, observational and descriptive study reviewed the skin tests results for indoor aeroallergens in pediatric patients (≤ 16 years) in 2011. We evaluated the results of skin tests specifically for: *Dermatophagoides farinae*, *Dermatophagoides pteronyssinus*, *Felis domesticus*, *Canis familiaris*, *Blattella germanica* and *Periplaneta americana*.

## Results

A total of 439 skin tests were performed for indoor aeroallergens in pediatric patients. There were 57.6% (n = 253) men and 42.4% (n = 186) women with mean age of 6.3 years. Patients were divided into the following age-groups: children under 3 years (17.8%, n = 78), 3 to 5 years (35%, n = 154), 6 to 12 years (36%, n = 158) and 13 to 16 years (11.2%, n = 49). The main diagnoses were chronic rhinopathy 88.9% (n = 390), asthma 16.7% (n = 73), atopic dermatitis 4.3% (n = 19) and other 7.3% (n = 32). At least 57.9% (n = 254) of the patients had one positive skin test for the evaluated allergens. In these patients, we found sensitization to *D. farinae* in 77.2% (n = 196), *D. pteronyssinus* 84.6% (n = 215), *B. germanica* 24% (n = 61), *P. American* 18.9% (n = 48), *F. domesticus* 18.5% (n = 47), and *C. familiaris *10.2% (n = 26).

## Conclusions

*D. farinae*, *D. pteronyssinus* and *B. germanica* were the most commonly aeroallergens found at the skin tests. When divided by age, *F. domesticus* and *C. familiaris* were more frequently found in children less than 3 year. In addition, *D. farinae* and *D. pteronyssinus* were identified more commonly in older age groups.

